# Movement Behaviors and Quality of Life in Cardiac Rehabilitation Programs: A Study Based on the Canadian 24‐Hour Movement Guidelines

**DOI:** 10.1002/pri.70281

**Published:** 2026-08-02

**Authors:** Júlio César de Ávila Soares, Maria Eduarda Martins Vigilato, Heloísa Balotari Valente, Alice Haniuda Moliterno, João Pedro Lucas Neves Silva, Diego Giulliano Destro Christofaro, Luiz Carlos Marques Vanderlei

**Affiliations:** ^1^ Department of Physiotherapy Faculty of Science and Technology São Paulo State University (UNESP) São Paulo Brazil; ^2^ Department of Physical Education Faculty of Science and Technology São Paulo State University (UNESP) São Paulo Brazil

**Keywords:** cardiac rehabilitation, physical activity, quality of life, sedentary behavior, sleep

## Abstract

**Background and Purpose:**

The Canadian 24‐Hour Movement Guidelines integrate recommendations on physical activity (PA), sedentary behavior (SB), and sleep. Individuals in cardiac rehabilitation programs (CRPs) have specific characteristics that may influence adherence to these 24‐Hour Movement Guidelines and their relationship with quality of life. This study aimed to investigate the proportion of CRP participants who meet the recommendations of the Canadian 24‐Hour Movement Guidelines and to evaluate the association between levels of PA, SB, and sleep duration with the quality‐of‐life domains of these individuals.

**Methods:**

This was a cross‐sectional observational study including CRP participants (mean age: 68.46 ± 10.15 years; 58.1% with coronary artery disease). Quality of life was assessed using the 36‐Item Short‐Form Health Survey (SF‐36). Movement behaviors (PA, SB, and sleep) were measured by accelerometers. Compliance with the guidelines was described using percentage values. Associations between PA, SB, and sleep with quality‐of‐life domains were analyzed using quantile regression. The significance level was set at 5%.

**Results:**

Among the 105 participants analyzed, only 2.9% simultaneously met all three recommendations. Individual adherence was 29.5% (PA), 9.5% (SB), and 34.3% (sleep). A negative association was identified between SB and functional capacity, and positive associations were found between PA and the domains of functional capacity, vitality, and social aspects. Sleep duration was not associated with quality‐of‐life domains.

**Discussion:**

Few CRP participants simultaneously meet all three recommendations of the Canadian 24‐Hour Movement Guidelines, with partial adherence being more frequent. Higher levels of PA and lower time spent in SB were associated with better quality of life, whereas sleep duration showed no significant association.

## 1 Introduction

Exercise‐based cardiac rehabilitation programs (CRPs) are a key strategy in the management of individuals with cardiovascular disease (CVD), providing clinical and functional benefits that have a significant impact on their quality of life (Dibben et al. 2023; Vieira et al. 2024). These programs aim to improve cardiorespiratory fitness, reduce risk factors, and promote the adoption of a healthier lifestyle, which decreases the recurrence of cardiovascular events and contributes to better patient adaptation to their clinical condition (Brown et al. 2024).

Although regular participation in CRPs is essential for the recovery and maintenance of overall health in individuals with CVD, the behaviors adopted by these individuals outside the supervised setting also play a crucial role in the rehabilitation process (Brown et al. 2024). Patients who, despite adhering to prescribed exercises, maintain a predominantly sedentary lifestyle and/or present inadequate sleep patterns may experience reduced functional gains (Santos et al. 2012; C. Tighe et al. 2020) and lower quality of life (Kim and Lee 2019; Hariri et al. 2024). Thus, assessing and intervening in these movement behaviors becomes essential to maximize the benefits of CRPs.

Therefore, a holistic approach that considers physical activity (PA), sedentary behavior (SB), and sleep concurrently—as proposed by the Canadian 24‐Hour Movement Guidelines—may offer a more comprehensive framework for optimizing outcomes in CRP participants. The 24‐Hour Movement Guidelines stand out by providing reference values to guide healthy behaviors throughout the day. In general, they emphasize the importance of 150 min per week of moderate‐to‐vigorous PA (MVPA), 7–9 h of sleep each night, and no more than 8 h of SB per day (Ross et al. 2020).

Although several studies have evaluated adherence to the Canadian 24‐Hour Movement Guidelines in the general population (Majed et al. 2024; Rollo et al. 2022; Riquelme et al. 2022; Chaput et al. 2014; Delfino et al. 2024; Rollo et al. 2020), evidence in clinical populations remains limited and has been concentrated mainly in pediatric samples, particularly among individuals with neurodevelopmental, behavioral, and mental health conditions (Duggan et al. 2025; Kong et al. 2023; Wang et al. 2022; Taylor et al. 2023; Zhao et al. 2024). Accordingly, there is a gap in the literature regarding CRP participants specifically. CRP participants represent a distinct clinical subgroup because, although they are exposed to structured and supervised exercise as part of secondary cardiovascular prevention, they may still accumulate high levels of sedentary time outside supervised sessions (Ramadi and Haennel 2019) and present inadequate sleep patterns (C. A. Tighe et al. 2022). In addition, this population commonly presents physical, functional, and psychological alterations that may affect movement behavior and well‐being (Komalasari and Nurjanah 2019). These factors may influence adherence to the recommendations as well as the relationship between PA, SB, sleep, and quality‐of‐life domains.

Considering the data described above, the present study aimed to: (1) investigate the proportion of CRP participants who meet each recommendation and the combined recommendations of the Canadian 24‐Hour Movement Guidelines; and (2) evaluate the associations between levels of PA, SB, and sleep duration with quality‐of‐life domains in CRP participants.

We hypothesized that less than 10% of CRP participants would meet all three Canadian 24‐Hour Movement Guidelines simultaneously. In addition, we expected that higher MVPA would be associated with better quality‐of‐life scores, particularly in the domains of physical functioning, physical role limitations, and bodily pain. Conversely, greater SB was expected to be associated with lower scores, mainly in physical functioning and physical role limitations. For sleep, we expected that longer sleep duration would be associated with better scores, especially in terms of mental health, vitality, and emotional role limitations. These associations were expected to persist after adjustment for demographic and clinical variables.

Identifying the proportion of CRP participants who meet the recommendations outlined in the Canadian 24‐Hour Movement Guidelines and understanding how levels of PA, SB, and sleep duration are associated with quality of life in these individuals are important for providing better support to educational programs that emphasize the importance of an active lifestyle and healthy sleep habits, thereby contributing to patients' adherence to these recommendations.

## 2 Methods

### 2.1 Population and Ethical Aspects

This cross‐sectional study was reported in accordance with the Strengthening the Reporting of Observational Studies in Epidemiology (STROBE) statement guidelines (Cuschieri 2019). Individuals diagnosed with CVD and/or risk factors were recruited from three different CRPs between May and November 2024 through consecutive face‐to‐face invitations conducted by an independent researcher with no connection to the participants. At the time of recruitment, participants had already been enrolled in the CRPs for varying lengths of time.

The three CRPs were selected by convenience and accessibility, as they represent all CRPs available in the municipality of Presidente Prudente, São Paulo, Brazil, where the study was conducted. These centers encompassed different institutional contexts, including a public service integrated with the Brazilian Unified Health System, a private cardiovascular rehabilitation center, and a university teaching clinic offering low‐cost community services. This approach allowed the inclusion of participants from different healthcare models and socioeconomic backgrounds. However, these centers should be interpreted as representative of the local CRP structure, rather than of Brazilian or international CRPs more broadly.

Participants who did not provide at least four valid days of accelerometer wear for the assessment of PA and SB (including three weekdays and one weekend day) (Monteiro et al. 2022) were excluded from the study. In addition, participants who did not achieve a minimum of seven valid days of sleep monitoring with the corresponding accelerometer were also excluded (Fekedulegn et al. 2020).

All procedures adopted in this research were previously approved by the Research Ethics Committee of the Faculty of Science and Technology—FCT/UNESP (CAAE: 78229424.0.0000.5402) on May 6, 2024, in accordance with the principles established in the Declaration of Helsinki (World Medical Association 2013). Prior to participation, volunteers received detailed information about the objectives and methods of the study and provided their consent by signing the informed consent form.

### 2.2 Experimental Procedure

The experimental procedures used in the study were divided into two distinct phases. In the first phase, information was collected for sample characterization, and participants had their quality of life assessed using the 36‐Item Short‐Form Health Survey (SF‐36). In the second phase, participants wore accelerometers to analyze movement patterns over the course of 1 week, including PA, SB, and sleep.

#### 2.2.1 First Stage of the Experimental Procedure

For sample characterization, participants' weight and height were measured using a digital scale (Plenna, TIN 00139 MAXIMA, Brazil) and a stadiometer (ES 2020—Sanny, Brazil). Based on these data, the body mass index (BMI) was calculated (Cercato et al. 2000). In addition, the following information was obtained from participants' medical records: sex, age, duration of participation in rehabilitation, primary clinical diagnosis, medication use, and presence of cardiovascular risk factors.

The cardiovascular risk factors considered included: family history (cases of coronary revascularization, acute myocardial infarction, or sudden death before the age of 55 in first‐degree male relatives or before the age of 65 in first‐degree female relatives); age (≥ 45 years for men and ≥ 55 years for women); hypertension [systolic blood pressure ≥ 140 mm of mercury (mmHg) or diastolic blood pressure ≥ 90 mmHg on two separate measurements, or use of antihypertensive medication]; dyslipidemia [low‐density lipoprotein (LDL) cholesterol > 130 mg per deciliter (mg/dL) or high‐density lipoprotein (HDL) cholesterol < 40 mg/dL on recent tests, or use of statins]; diabetes mellitus (fasting blood glucose ≥ 100 mg/dL, confirmed by two separate measurements, or use of hypoglycemic agents or insulin); and smoking (current smokers or individuals who quit the habit less than six months prior) (American College of Sports Medicine 2007).

#### 2.2.2 Quality of Life

To assess participants' quality of life, the SF‐36 questionnaire was used, which has been adapted and validated for Brazilian Portuguese (Laguardia et al. 2013). This questionnaire consists of 36 items addressing eight dimensions of quality of life: physical functioning, physical role limitations, bodily pain, general health condition, vitality, social functioning, emotional role limitations, and mental health. Responses are scored on a raw scale from 0 to 100 for each dimension, where 0 represents the worst and 100 represents the best quality of life (Ware and Sherbourne 1992).

#### 2.2.3 Second Stage of the Experimental Procedure

Assessment of SB and PA levels was carried out using a triaxial accelerometer, ActiGraph GT3X‐BT (ActiGraph LLC, Pensacola, FL, USA), over seven consecutive days. Participants were instructed to wear the device on the right hip using an elastic belt and to keep it on throughout the day, removing it only for sleeping or during water‐based activities such as bathing and swimming. To complement data collection, volunteers completed a diary recording the times they went to bed, woke up, and bathed during the monitoring period (Chomistek et al. 2017).

After data collection, the records were processed in 60‐s epochs using ActiLife software version 6.13.4 (ActiGraph LLC, Pensacola, FL, USA) (Whitaker et al. 2018). Only records with a minimum of 10 h of daily wear time, on at least three weekdays and one weekend day, were considered valid. Non‐wear time of the accelerometer was defined as periods of at least 60 consecutive minutes of zero activity counts, and these periods were excluded from daily wear time (Chomistek et al. 2017). SB was defined using a cut‐off point of ≤ 100 counts per minute (cpm), while MVPA was defined as ≥ 2020 cpm (Dempsey et al. 2020). Participants with SB ≤ 8 h per day and MVPA ≥ 150 min per week were classified as meeting the Canadian 24‐Hour Movement Guideline (Ross et al. 2020). For the association of SB and MVPA with quality‐of‐life domains, these variables were expressed in minutes/day and as a percentage of time. In addition, daily steps and steps per minute were also considered as PA outcomes.

For sleep assessment, a second accelerometer was placed on the participants' non‐dominant wrist during the same 7 days of PA and SB monitoring. In this case, the device could only be removed for water‐based activities. Participants also recorded in a diary their wake‐up time, time out of bed, naps, bedtime, sleep onset, and any moment when they removed the accelerometer (Meltzer and Westin 2011).

To estimate sleep duration, wrist accelerometer data were analyzed using ActiLife software version 6.13.4 (ActiGraph LLC, Pensacola, FL, USA). Sleep windows were identified through visual inspection of actograms for all participants, considering the interval between the interruption and resumption of PA. Sleep diary information, including reported bedtime, sleep onset, wake‐up time, time out of bed, naps, and device removal, was used only as supportive information to assist this process when available. Diary completion was not used as an exclusion criterion, and no participant was excluded due to inadequate or incomplete diary completion (Sadeh et al. 1994). No subjective measure of sleep quality was applied; therefore, sleep analyses were restricted to sleep duration.

The number of participants who met the sleep duration recommended by the Canadian 24‐Hour Movement Guideline was calculated (7–9 h/night for individuals aged 18–64 years and 7–8 h/night for individuals ≥ 65 years) (Ross et al. 2020). For the association between sleep duration and quality‐of‐life domains, this variable was expressed in minutes/night. To ensure correct use of the accelerometers on the hip and wrist, all participants received detailed written instructions. In addition, the research team reinforced the instructions through daily reminders throughout the monitoring period, ensuring that the usage protocols were properly followed.

### 2.3 Statistical Analysis

The sample size was determined based on the correlation coefficient between SB (measured by accelerometer) and the general health domain of the SF‐36 questionnaire, using as reference a pilot study with 12 participants. An estimated correlation of −0.382, an alpha error of 5%, and a statistical power of 80% were considered, resulting in a minimum required sample size of 51 participants. Because the adjusted analyses included up to five covariates, an additional 50 participants were planned as a strategy to support adjusted analyses, considering commonly used sample size recommendations for regression models (Field 2009). Therefore, the target sample size was increased to 101 participants. The sample size calculation was performed using MedCalc Software bvba—version 19.2.6 (Ostend, Belgium).

Descriptive statistics were used to characterize the sample and outcomes, with results presented as mean, standard deviation, median, interquartile range, absolute frequency, and percentage. Normality of the data was assessed using the Shapiro‐Wilk test.

To compare the proportions of men and women meeting each of the guidelines individually (MVPA, SB, and sleep), the chi‐square test was used. The same analysis was also applied to compare the distribution of men and women regarding the number of guidelines met, categorized as: none, only one guideline (regardless of which), two guidelines (regardless of which), and all three guidelines.

In addition, to compare mean age and BMI among groups of participants classified according to the number of movement guidelines met (none, one, two, or three), a one‐way analysis of variance (ANOVA) was conducted, followed by Tukey's post hoc test. Homogeneity of the data was assessed using Levene's test.

To examine the associations of SB, PA, and sleep with quality‐of‐life domains, quantile regression was employed due to the non‐normal distribution of SF‐36 scores, as this approach provides robust estimates under this condition (Khadka et al. 2024). The 0.50 quantile, corresponding to the median, was modeled because the objective was to estimate associations with the central tendency of the quality‐of‐life domains. No additional quantiles were tested.

Initially, Model 1 (unadjusted) was applied, and, according to the significance of the results, additional models were used: Model 2 (adjusted for sex and age), Model 3 (adjusted for sex, age, and rehabilitation duration), Model 4 [adjusted for sex, age, rehabilitation duration, and presence of comorbidities (hypertension, dyslipidemia, and diabetes mellitus)], and model 5 (adjusted for sex, age, rehabilitation duration, and use of lipid‐lowering agents and beta‐blockers). The adjustment variables were selected a priori as potential confounders based on clinical plausibility and previous literature investigating movement behaviors and health‐related quality‐of‐life outcomes in cardiac populations, as age, sex, rehabilitation duration, comorbidities, and medication use may influence participants' movement patterns, functional status, clinical condition, and perceived quality of life (Wardoku et al. 2019; Freene et al. 2022). The significance level was set at 5%. Analysis was performed using the Statistical Package for the Social Sciences (SPSS), version 30.0 (SPSS Inc., Chicago, IL, USA).

## 3 Results

**FIGURE 1:**
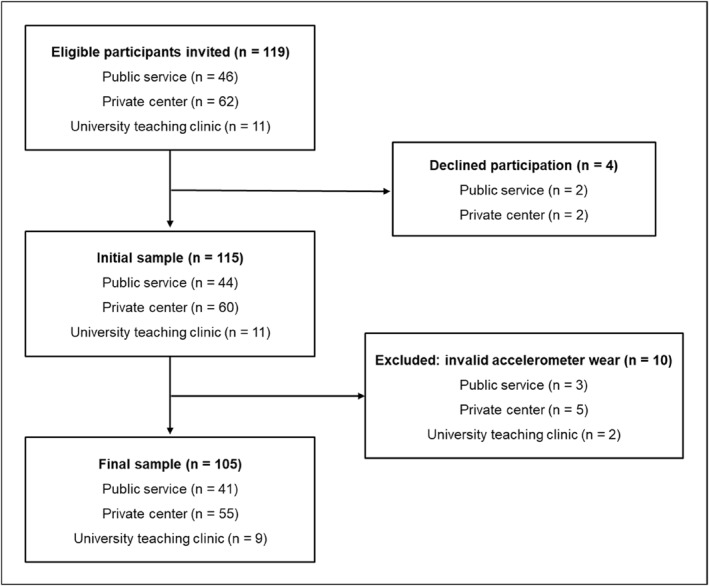
Sample flowchart. *n* = number of participants.

Figure 1 shows the sample flowchart. Initially, 119 eligible CRP participants were invited to participate: 46 from the public service, 62 from the private center, and 11 from the university teaching clinic. Four individuals declined participation, resulting in an initial sample of 115 participants. After excluding 10 participants for inadequate accelerometer wear, the final sample consisted of 105 individuals: 41 from the public service, 55 from the private center, and nine from the university teaching clinic. Of these, 56 were men (53.3%) and 49 were women (46.7%).

The information regarding sample characterization is presented in Table 1. The population was predominantly older adults, with a mean age of 68.46 ± 10.15 years, and overweight [mean BMI of 28.87 ± 4.49 kg per square meter (kg/m^2^)]. Most participants had been in the program for more than 1 year (54.3%). The primary clinical diagnosis was coronary artery disease (58.1%), and the main risk factors identified were age (93.3%), hypertension (83.8%), and dyslipidemia (76.2%). Medication use was frequent, with lipid‐lowering agents (76.2%) and beta‐blockers (59.0%) being the most common.

**TABLE 1:** Sample characterization.

Variables	*n* = 105
Age (years)	68.46 ± 10.15 (69.00) [63.00–76.00]
Weight (kg)	76.47 ± 15.81 (75.95) [64.60–85.50]
Height (m)	1.62 ± 0.10 (1.63) [1.55–1.69]
BMI (kg/m^2^)	28.87 ± 4.49 (27.67) [25.98–30.96]
Sex, *n* (%)	
Men	56 (53.3)
Women	49 (46.7)
Length of participation in the CRP, *n* (%)	
Up to 3 months	26 (24.8)
Over 3 up to 6 months	6 (5.7)
Over 6 months up to 1 year	16 (15.2)
Over 1 year	57 (54.3)
Main clinical diagnosis, *n* (%)	
Coronary artery disease	61 (58.1)
Other diseases	35 (33.3)
Primary prevention	9 (8.6)
Cardiovascular risk factors, *n* (%)	
Age	98 (93.3)
Hypertension	88 (83.8)
Dyslipidemia	80 (76.2)
Family history	69 (65.7)
Diabetes mellitus	40 (38.1)
Smoking	3 (2.9)
Medicines, *n* (%)	
Lipid‐lowering agents	80 (76.2)
Beta blockers	62 (59.0)
ARA II	56 (53.3)
Antiplatelet agents	50 (47.6)
Hypoglycemic agents	40 (38.1)
Diuretics	34 (32.4)
ACE inhibitors	17 (16.2)
Vasodilators	15 (14.3)
Others	36 (34.3)

*Note:* Values expressed as: mean ± standard deviation (median) [interquartile range (25%–75%)]; absolute number (percentage values).

Abbreviations: %: percentage; ACE: angiotensin‐converting enzyme; ARA II: angiotensin II receptor antagonists; BMI: body mass index; CRP: cardiac rehabilitation program; kg: kilograms; kg/m^2^: kilogram per meter squared; m: meters; *n*: number of participants; Other diseases: cardiomyopathies, rhythm disorders, valvular heart disease, heart failure, and congenital heart diseases.

Table 2 presents the descriptive statistics of the independent and dependent variables of the study, including adherence to the Canadian 24‐Hour Movement Guidelines. Accelerometry analysis revealed a mean of 598.40 ± 82.63 min/day (9.97 h/day) in SB, while MVPA time was 16.97 ± 15.10 min/day (118.79 min/week). The mean sleep duration was 413.15 ± 64.79 min/night (6.89 h/night).

**TABLE 2:** Descriptive statistics of the independent and dependent variables of the study and adherence to movement guidelines.

Variables	*n* = 105
Accelerometry	
Sedentary time (min/day)	598.40 ± 82.83 (600.00) [550.25–648.75]
Percentage of time in SB	63.74 ± 8.53 (64.67) [57.89–69.56]
MVPA (min/day)	16.97 ± 15.10 (11.75) [6.00–24.00]
Percentage of time in MVPA	1.96 ± 1.74 (1.46) [0.71–2.71]
Steps per day	5665.21 ± 2326.71 (5355.50) [3843.25–7324.75]
Steps per minute	6.54 ± 2.55 (6.28) [4.50–8.45]
Sleep (min/night)	413.15 ± 64.79 (411.43) [372.86–453.86]
Adherence to the guidelines, *n* (%)	
MVPA	31 (29.5)
SB	10 (9.5)
Sleep	36 (34.3)
Degree of adherence to the guidelines, *n* (%)	
No guideline	44 (41.9)
One guideline	48 (45.7)
Two guidelines	10 (9.5)
Three guidelines	3 (2.9)
Quality‐of‐life domains (SF‐36)	
Physical functioning	74.52 ± 18.93 (80.00) [60.00–90.00]
Physical role limitations	70.71 ± 38.21 (100.00) [25.00–100.00]
Bodily pain	65.39 ± 21.60 (62.00) [51.00–90.00]
General health condition	68.87 ± 18.17 (72.00) [52.00–82.00]
Vitality	63.10 ± 17.70 (65.00) [50.00–75.00]
Social functioning	79.05 ± 21.68 (87.50) [62.50–100.00]
Emotional role limitations	80.95 ± 34.24 (100.00) [66.67–100.00]
Mental health	73.49 ± 17.11 (76.00) [60.00–88.00]

*Note:* Values expressed as: mean ± standard deviation (median) [interquartile range (25%–75%)]; absolute number (percentage values). Adherence to the Canadian 24‐Hour Movement Guidelines was defined as MVPA ≥ 150 min/week, SB ≤ 8 h/day, and sleep duration of 7–9 h/night for adults aged 18–64 years and 7–8 h/night for adults aged ≥ 65 years.

Abbreviations: %: percentage; min: minutes; MVPA: moderate to vigorous physical activity; *n*: number of participants; SB: sedentary behavior; SF‐36: Short Form Health Survey 36.

As shown in Table 2, overall adherence to the movement guidelines was low. Only 29.5% of participants met the MVPA recommendation, 9.5% met the SB recommendation, and 34.3% achieved the recommended sleep duration. Regarding simultaneous adherence, 41.9% of participants did not meet any guideline, 45.7% met one guideline, 9.5% met two guidelines, and only 2.9% met all three recommendations.

Regarding the SF‐36 questionnaire assessment, as shown in Table 2, participants showed higher scores in the domains of emotional role (80.95 ± 34.24), social functioning (79.05 ± 21.68), and physical functioning (74.52 ± 18.93), whereas vitality (63.10 ± 17.70) and bodily pain (65.39 ± 21.60) were the domains with the lowest quality‐of‐life scores.

**FIGURE 2:**
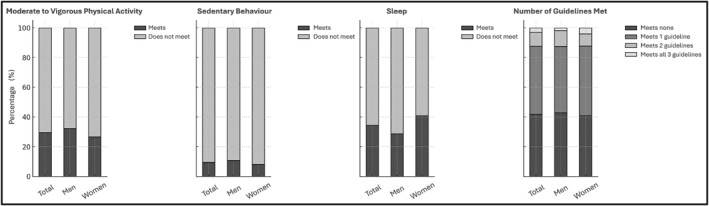
Percentage distribution of participants meeting each recommendation of the Canadian 24‐Hour Movement Guidelines according to sex, as well as the number of guidelines met simultaneously (none, one, two, or three).

As illustrated in Figure 2, the distribution of participants meeting each movement guideline did not differ significantly between men and women: MVPA (women: 26.5%; men: 32.1%; *p* = 0.529), SB (women: 8.2%; men: 10.7%; *p* = 0.748), and sleep duration (women: 40.8%; men: 28.6%; *p* = 0.187). Figure 2 also shows that the distribution of participants according to the number of recommendations met simultaneously did not differ significantly between sexes: no guideline (women: 40.8%; men: 42.9%; *p* = 0.833), one guideline (women: 46.9%; men: 44.6%; *p* = 0.814), two guidelines (women: 8.2%; men: 10.7%; *p* = 0.748), and all three guidelines (women: 4.1%; men: 1.9%; *p* = 0.597).

**FIGURE 3:**
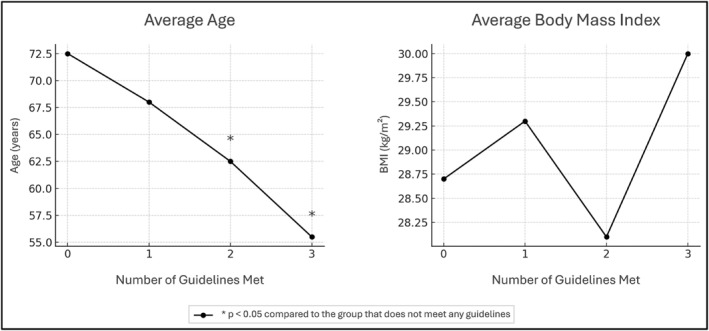
Average values of age and body mass index of participants according to the number of Canadian 24‐Hour Movement Guidelines met. Age differed between groups (ANOVA: *p* = 0.003), but body mass index did not (ANOVA: *p* = 0.803). Tukey's post hoc: no guideline versus two guidelines, *p* = 0.029; no guideline versus all three guidelines, *p* = 0.031. No other pairwise comparisons were statistically significant.

As illustrated in Figure 3, mean age and BMI varied according to the number of movement guidelines met. A trend of decreasing mean age was observed as the number of guidelines met increased. ANOVA indicated a statistically significant difference between groups [*F* (3, 101) = 4.960; *p* = 0.003], with Tukey's post hoc test revealing that individuals who did not meet any guideline were older (71.7 ± 8.2 years) than those who met two (62.2 ± 14.2 years; *p* = 0.029) or three recommendations (55.7 ± 20.4 years; *p* = 0.031). In contrast, as also shown in Figure 3, no significant differences were observed in BMI values between the groups [*F* (3, 101) = 0.329; *p* = 0.803].

The simple quantile regression model assessing the association of SB, PA, and sleep variables with quality‐of‐life domains is presented in Table 3. Negative associations were observed between SB and the physical functioning domain (*β*: −0.070; *p* = 0.019 for minutes per day in SB; *β*: −0.877; *p* = 0.002 for percentage of time in SB). In contrast, positive associations were found between MVPA variables (expressed in minutes per day and percentage of time) and the domains of physical functioning, vitality, and social functioning (*p* < 0.05). Only MVPA in minutes per day was positively associated with the pain domain (*β*: 0.502; *p* = 0.028).

**TABLE 3:** Association of sedentary behavior, physical activity and sleep variables with quality‐of‐life domains according to the 36‐Item Short‐Form Health Survey (SF‐36).

Variables	Medical outcomes study 36‐Item Short‐Form Health Survey (SF‐36)							
	Physical functioning	Physical limitations	Bodily pain	General health condition	Vitality	Social functioning	Emotional limitations	Mental health
Sedentary behavior (min/day)	**−0.070 (−0.128; −0.012)** ***p* = 0.019**	5.623 (−0.173; 0.173) *p* = 1.000	0.049 (−0.036; 0.135) *p* = 0.254	8.056 (−0.069; 0.069) *p* = 1.000	−3.793 (−0.058; 0.058) *p* = 1.000	−2.776 (−0.086; 0.086) *p* = 1.000	1.581 (−0.077; 0.077) *p* = 1.000	0.018 (−0.044; 0.080) *p* = 0.573
Sedentary behavior (%)	**−0.877 (−1.416; −0.338)** ***p* = 0.002**	−8.263 (−1.660; 1.660) *p* = 1.000	0.552 (−0.189; 1.294) *p* = 0.143	1.110 (−0.664; 0.664) *p* = 1.000	−0.371 (0.920; 0.178) *p* = 0.183	−0.360 (−1.163; 0.444) *p* = 0.377	−1.804 (−0.738; 0.738) *p* = 1.000	0.291 (−0.268; 0.849) *p* = 0.291
MVPA (min/day)	**0.548 (0.223; 0.873)** ***p* = 0.001**	3.600 (−0.941; 0.941) *p* = 1.000	**0.502 (0.054; 0.951)** ***p* = 0.028**	1.386 (−0.376; 0.376) *p* = 1.000	**0.296 (0.024; 0.569)** ***p* = 0.033**	**0.435 (0.041; 0.829)** ***p* = 0.031**	6.966 (−0.418; 0.418) *p* = 1.000	0.164 (−0.163; 0.491) *p* = 0.322
MVPA (%)	**4.931 (2.212; 7.650)** ***p* = 0.000**	1.545 (−8.179; 8.179) *p* = 1.000	3.613 (−0.370; 7.597) *p* = 0.075	4.031 (−3.272; 3.272) *p* = 1.000	**3.040 (0.703; 5.376)** ***p* = 0.011**	**3.840 (0.259; 7.422)** ***p* = 0.036**	9.264 (−3.635; 3.635) *p* = 1.000	1.479 (−1.346; 4.303) *p* = 0.302
Steps per day	**0.004 (0.002; 0.006)** ***p* = 0.000**	0.003 (−0.003; 0.009) *p* = 0.292	0.002 (−0.001; 0.005) *p* = 0.272	0.001 (−0.001; 0.003) *p* = 0.369	0.002 (0.000; 0.003) *p* = 0.070	**0.003 (0.001; 0.006)** ***p* = 0.016**	−2.875 (−0.003; 0.003) *p* = 1.000	2.168 (−0.002; 0.002) *p* = 1.000
Steps per minute	**3.468 (1.756; 5.181)** ***p* = 0.000**	2.688 (−2.190; 7.566) *p* = 0.277	1.753 (−0.711; 4.217) *p* = 0.161	5.910 (−2.211; 2.211) *p* = 1.000	**1.942 (0.289; 3.594)** ***p* = 0.022**	2.326 (−0.206; 4.858) *p* = 0.071	−2.808 (−2.456; 2.456) *p* = 1.000	9.141 (−2.063; 2.063) *p* = 1.000
Sleep (min/night)	0.037 (−0.046; 0.121) *p* = 0.375	−5.931 (−0.218; 0.218) *p* = 1.000	0.030 (−0.079; 0.138) *p* = 0.588	0.049 (−0.033; 0.131) *p* = 0.241	−4.375 (−0.073; 0.073) *p* = 1.000	−0.059 (−0.159; 0.041) *p* = 0.247	−1.553 (−0.097; 0.097) *p* = 1.000	−0.043 (−0.120; 0.033) *p* = 0.264

*Note:* Values expressed as: coefficient (95% confidence interval) *p*‐value. Bold: significant values.

Abbreviations: %: percentage of time using the accelerometer; min: minutes; MVPA: moderate to vigorous physical activity.

Steps per day were also positively associated with the domains of physical functioning (*β*: 0.004; *p* = 0.000) and social functioning (*β*: 0.003; *p* = 0.016), and steps per minute with the domains of physical functioning (*β*: 3.468; *p* = 0.000) and vitality (*β*: 1.942; *p* = 0.022). No significant associations were identified between sleep duration and quality‐of‐life domains.

Table 4 presents the quantile regression models considering the adjustment variables age, sex, rehabilitation duration, presence of comorbidities (hypertension, dyslipidemia, and diabetes mellitus), and use of lipid‐lowering agents and beta‐blockers. Except for SB in minutes per day (*β*: −0.051; *p* = 0.065 in Model 2) and MVPA in minutes per day (*β*: 0.321; *p* = 0.061 in Model 5), all other significant associations with physical functioning were maintained regardless of the adjustment model. The same was observed for the percentage of time in MVPA and steps per minute with vitality (*p* < 0.05).

**TABLE 4:** Association of sedentary behavior and physical activity variables with quality‐of‐life domains according to the 36‐Item Short‐Form Health Survey (SF‐36) in adjusted quantile regression models.

Variables		Medical outcomes study 36‐Item Short‐Form Health Survey (SF‐36)			
		Physical functioning	Bodily pain	Vitality	Social functioning
Sedentary behavior (min/day)	Model 2	−0.051 (−0.106; 0.003) *p* = 0.065	—	—	—
Sedentary behavior (%)	Model 2	**−0.966 (−1.568; −0.364) *p* = 0.002**	—	—	—
	Model 3	**−1.010 (−1.637; −0.382) *p* = 0.002**	—	—	—
	Model 4	**−0.783 (−1.369; −0.197) *p* = 0.009**	—	—	—
	Model 5	**−0.766 (−1.347; −0.184) *p* = 0.010**	—	—	—
MVPA (min/day)	Model 2	**0.455 (0.147; 0.762) *p* = 0.004**	**0.500 (0.075; 0.925) *p* = 0.021**	0.298 (−0.017; 0.614) *p* = 0.063	0.296 (−0.172; 0.764) *p* = 0.213
	Model 3	**0.455 (0.146; 0.764) *p* = 0.004**	0.418 (−0.003; 0.838) *p* = 0.052	—	—
	Model 4	**0.520 (0.202; 0.839) *p* = 0.002**	—	—	—
	Model 5	0.321 (−0.015; 0.657) *p* = 0.061	—	—	—
MVPA (%)	Model 2	**4.050 (1.403; 6.696) *p* = 0.003**	—	**3.739 (1.148; 6.329) *p* = 0.005**	2.605 (−1.447; 6.658) *p* = 0.205
	Model 3	**4.418 (1.687; 7.149) *p* = 0.002**	—	**3.965 (1.491; 6.439) *p* = 0.002**	—
	Model 4	**4.500 (1.806; 7.193) *p* = 0.001**	—	**3.413 (1.034; 5.792) *p* = 0.005**	—
	Model 5	**2.990 (0.082; 5.898) *p* = 0.044**	—	**4.221 (1.902; 6.540) *p* = 0.000**	—
Steps per day	Model 2	**0.004 (0.003; 0.006) *p* = 0.000**	—	—	**0.003 (0.001; 0.006) *p* = 0.016**
	Model 3	**0.004 (0.003; 0.006) *p* = 0.000**	—	—	**0.003 (6.238; 0.005) *p* = 0.045**
	Model 4	**0.004 (0.002; 0.006) *p* = 0.000**	—	—	**0.003 (0.000; 0.005) *p* = 0.034**
	Model 5	**0.004 (0.002; 0.006) *p* = 0.000**	—	—	0.001 (−0.002; 0.004) *p* = 0.416
Steps per minute	Model 2	**4.368 (2.875; 5.861) *p* = 0.000**	—	**2.057 (0.264; 3.849) *p* = 0.025**	—
	Model 3	**4.361 (2.863; 5.858) *p* = 0.000**	—	**2.043 (0.302; 3.784) *p* = 0.022**	—
	Model 4	**4.293 (2.733; 5.853) *p* = 0.000**	—	**2.355 (0.589; 4.121) *p* = 0.009**	—
	Model 5	**4.134 (2.481; 5.786) *p* = 0.000**	—	**2.092 (0.345; 3.839) *p* = 0.019**	—

*Note:* Values expressed as: coefficient (95% confidence interval) *p*‐value. Bold: significant values. Model 2: quantile regression adjusted for age and sex. Model 3: model 2 + adjustment for rehabilitation time. Model 4: model 3 + adjustment for presence of comorbidities (hypertension, dyslipidemia, and diabetes mellitus). Model 5: model 3 + adjustment for use of lipid‐lowering agents and beta‐blockers.

Abbreviations: %: percentage of accelerometer use time; min: minutes; MVPA: moderate to vigorous physical activity.

The associations observed between MVPA in minutes per day and the domains of vitality (*β*: 0.298; *p* = 0.063) and social functioning (*β*: 0.296; *p* = 0.213) were lost in Model 2, which also occurred for the association between percentage of time in MVPA and the social functioning domain (*β*: 2.605; *p* = 0.205). The association between MVPA in minutes per day and the pain domain was lost in Model 3 (*β*: 0.418; *p* = 0.052). In addition, the association between steps per day and social functioning was lost in Model 5 (*β*: 0.001; *p* = 0.416).

## 4 Discussion

The results of this study indicate that most CRP participants meet at least one of the Canadian 24‐Hour Movement Guidelines (45.7%), but few fully adhere to all recommendations (2.9%). In addition, SB was negatively associated with the physical functioning domain, whereas PA variables showed positive associations with some quality‐of‐life domains, such as physical functioning and vitality. Sleep duration did not show a significant relationship with quality‐of‐life domains among the study participants.

These findings are consistent with previous studies that point to low adherence to the Canadian 24‐Hour Movement Guidelines in their entirety. National estimates from Canada indicate that fewer than 10% of Canadian adults simultaneously achieve all three recommendations (Rollo et al. 2022). Among older adults, full adherence is even lower, dropping to around 1.8%, as demonstrated in a Chinese study (Liang et al. 2024). Similar to our study, this evidence shows that most people meet only one or two recommendations (Rollo et al. 2022; Liang et al. 2024), highlighting that partial compliance is common, whereas full adherence is rare.

These findings suggest that adequately integrating the three movement behaviors into daily life is a significant challenge, even among participants engaged in CRPs, for whom low adherence may reflect barriers such as physical limitations, low confidence in modifying daily routines, insufficient knowledge about the risks of prolonged SB, and limited opportunities or support to incorporate movement throughout the day outside supervised sessions (Yang et al. 2025).

With regard to sex, no statistically significant differences were observed between men and women in meeting the recommendations. However, the percentage variations observed, although not reaching statistical significance, follow a pattern consistent with the literature, which often reports greater male engagement in vigorous PA (The Lancet Public Health 2019), whereas women tend to exhibit better sleep habits (Bixler et al. 2009).

In contrast, age appeared to be associated with adherence to the movement recommendations, with younger participants meeting a greater number of guidelines. This is consistent with population‐based studies indicating poorer adherence among older adults, especially to PA and SB recommendations (Liang et al. 2024). This finding may be related, at least in part, to functional decline associated with aging and the higher prevalence of comorbidities, which may make it more difficult to achieve the recommended levels of PA (Castellanos‐Perilla et al. 2020).

In our sample, BMI did not show a clear pattern of association with the degree of adherence to movement recommendations, in contrast to previous studies that reported lower adherence among individuals with overweight or obesity (Baillot et al. 2022). A possible explanation for our findings is the specificity of the sample; that is, all participants were enrolled in CRPs and therefore may have been motivated to adopt lifestyle changes regardless of BMI. This is a positive aspect, given the importance of engaging patients with obesity in PA and reducing SB.

Regarding SB, it showed a negative association with physical functioning, a finding that aligns with the literature, which indicates that prolonged periods of SB are related to reduced physical function and poorer performance in chair rise and walking tests, especially among older adults (Gilchrist et al. 2022). Potential mechanisms, such as muscle atrophy and reduced cardiorespiratory capacity related to prolonged sedentary time, may help explain this relationship (Silva et al. 2020).

In contrast to SB, higher levels of PA were positively associated with quality‐of‐life domains, such as physical functioning and vitality. These findings are consistent with the literature, which suggests that physically active individuals tend to have greater vitality and better physical function compared with their sedentary counterparts (Marquez et al. 2020). These associations may be related to better cardiorespiratory fitness and muscle strength observed among individuals with higher PA levels (Brellenthin et al. 2022).

Unlike PA and SB parameters, sleep duration was not significantly associated with quality‐of‐life domains in our analysis. This finding may seem counterintuitive given the recognized importance of sleep for health (Ramar et al. 2021); however, it may be explained by methodological, sample‐related, and clinical factors. First, our study assessed sleep only in terms of duration and did not evaluate other sleep dimensions, such as quality, efficiency, nocturnal awakenings, timing, or sleep‐wake regularity, which may be more closely related to health‐related quality of life (DelRosso 2025; Kohyama 2021; Kudrnáčová and Kudrnáč 2023; Silay and Gursoy 2024).

Second, most participants in the present study had sleep duration within a near‐normal range, with no marked sleep deprivation or excessive sleep duration. This characteristic may have reduced the likelihood of observing significant associations with quality‐of‐life domains. This interpretation is consistent with longitudinal evidence suggesting that more pronounced impairments in physical and mental quality of life are mainly observed at extreme sleep durations, such as very short sleep (≤ 5 h) or very long sleep (≥ 10 h), whereas moderate differences in sleep duration appear to have less impact (Faubel et al. 2009). In addition, unmeasured clinical factors, such as sleep‐disordered breathing, chronic pain, depressive symptoms, or other comorbid conditions not captured in the adjusted models, may also have influenced the relationship between sleep duration and quality of life (Nistor et al. 2023).

Among the limitations of this study, the cross‐sectional design stands out, as it prevents causal inference and does not allow determination of the directionality of the associations observed between movement behaviors and quality‐of‐life outcomes. For example, we cannot determine whether lower SB contributes to better physical functioning or whether individuals with better physical functioning tend to spend less time in SB. In addition, participants were recruited by convenience from CRPs located in a single urban municipality, which may limit the external validity of the findings and their generalizability to other regions, healthcare systems, and cultural contexts.

Another limitation relates to the use of accelerometers to assess PA and SB. Although accelerometry provided objective measures of movement, it may have underestimated some activities, such as cycling, resistance training, and water‐based or upper‐body movements. In addition, the use of a single cutoff to define MVPA (≥ 2020 cpm) may not fully capture moderate‐intensity activities in older or deconditioned adults, for whom lower absolute movement counts may still represent relevant physiological effort. Moreover, although participants wore an accelerometer during supervised CRP sessions, exercise intensity during these sessions was not analyzed separately from daily movement, limiting our ability to isolate the specific contribution of the rehabilitation program itself.

Regarding sleep assessment, our analysis focused primarily on accelerometry‐derived duration, without subjective measures of sleep quality or additional information on insomnia, sleep fragmentation, sleep timing, sleep regularity, or sleep‐disordered breathing. Finally, although the analyses were adjusted for the two medication classes most frequently used by the sample, lipid‐lowering agents and beta‐blockers, other medication classes and additional variables, such as educational level and socioeconomic status, were not included in the adjusted models and may have influenced movement behaviors and quality‐of‐life outcomes. All these factors should be considered when interpreting the findings.

Given the limitations mentioned above, future studies may adopt longitudinal designs to investigate how changes in movement behaviors over time are related to quality of life in CRP participants. In addition, studies involving multicenter, nationwide, or international samples are needed to confirm these findings and to improve external validity. Furthermore, the use of statistical approaches that consider behaviors in an integrated manner is recommended, allowing for a more realistic analysis aligned with the Canadian 24‐Hour Movement Guidelines.

Future research should also consider combining accelerometry with complementary assessment methods (e.g., self‐reported measures or activity logs) to better capture different types of PA that may not be fully detected by wearable devices. Moreover, studies should incorporate both objective and subjective sleep measures, including validated sleep quality questionnaires, to better characterize the multidimensional nature of sleep and its relationship with quality of life.

In conclusion, few CRP participants simultaneously meet all three recommendations described in the Canadian 24‐Hour Movement Guidelines, with partial adherence to the guidelines being more common. A negative association was observed between SB and the physical functioning domain of the SF‐36 questionnaire, and positive associations were found between PA and quality‐of‐life domains, including physical functioning and vitality. Sleep duration was not significantly associated with quality of life in the participants. These findings suggest that adequate PA levels and lower SB are relevant behavioral targets in CRPs, as they were associated with better quality‐of‐life profiles in this population.

### 4.1 Implications of Physiotherapy Practice

Taken together, the findings of this study have important clinical implications for CRPs. The low simultaneous adherence to PA, SB, and sleep recommendations highlights the need for interventions that address broader and more integrated behavioral changes. Furthermore, the associations found between higher PA levels and lower SB with better quality‐of‐life scores suggest that these components should receive attention in cardiac patient care.

These results suggest that CRPs should incorporate strategies aimed at increasing engagement in PA and reducing sedentary time throughout the day, not only during supervised sessions but also in the home setting. Such strategies may include encouraging regular interruptions of prolonged sedentary time, such as standing up and walking for 2–3 min every 30 min of sitting, promoting light‐intensity PA integrated into daily routines, and applying behavior change techniques, such as step‐count goals, daily self‐monitoring, and individualized counseling, with the aim of supporting long‐term adherence. In addition, identifying more vulnerable subgroups, such as older individuals with lower adherence to the guidelines, may guide more individualized actions within the programs.

## Funding

This work was supported by the Coordenação de Aperfeiçoamento de Pessoal de Nível Superior—Brasil (CAPES)—Finance Code 001.

## Ethics Statement

All procedures adopted in this research were previously approved by the Research Ethics Committee of the Faculty of Science and Technology—FCT/UNESP (CAAE: 78229424.0.0000.5402) on May 6, 2024, following the principles established in the Declaration of Helsinki.

## Consent

Prior to participation, volunteers received detailed information about the objectives and methods of the study and provided their consent by signing the informed consent form.

## Conflicts of Interest

The authors declare no conflicts of interest.

## Data Availability

The data that support the findings of this study are available from the corresponding author upon reasonable request.
